# Authors Reply to 'Strategies for Overcoming T-Wave Oversensing'

**DOI:** 10.1016/s0972-6292(16)30824-5

**Published:** 2014-12-15

**Authors:** Becker SN Alzand, TJE Phlips, Rik Willems

**Affiliations:** Department of Cardiology, Glorieux General Hospital, Ronse, Belgium

**Keywords:** T-Wave Oversensing

In their letter to the editor Cay et al [1] express some valid concerns regarding the strategy we described for overcoming T-wave oversensing in our patient [2]. They stress that programmable features of the device should be used first before planning a reintervention. As mentioned in our case-report we tried both the enhanced T wave suppression algorithm available in the device and programming a low sensitivity of 0.9 mv but T-wave oversensing remained. They also stated that changing ventricular sensing configuration can result in higher R-wave amplitude (>3 mV) and loss of T-wave sensing. Changing ventricular sensing configuration is an option in pacemakers (bipolar to unipolar) and some ICDs (true bipolar to integrated bipolar) but unfortunately this option was not available in the device used in our patient. Another suggestion was to change the timing and sequence of ventricular pacing with various V-V pace delay settings to solve the problem of TWOS. Changing the initially chosen optimal V-V interval is often not a practical solution as it might lead to broader QRS complex and loss of optimal synchronization leading to LV function deterioration. The last possible solution they suggest was to increase therapy zones to high ranges and detection intervals to prevent delivery of inappropriate therapies. As they also stated this is inappropriate as the V-V interval during T wave oversensing were about 220 ms. Finally they proposed to perform repositioning of the defibrillator lead before implanting a new lead, but as they realize this it is a more complex procedure. We thank them for their correction of our figures.
